# Dissecting Time- from Tumor-Related Gene Expression Variability in Bilateral Breast Cancer

**DOI:** 10.3390/ijms19010196

**Published:** 2018-01-09

**Authors:** Maurizio Callari, Matteo Dugo, Patrizia Miodini, Silvia Veneroni, Giampaolo Bianchini, Maria Grazia Daidone, Vera Cappelletti

**Affiliations:** 1 Department of Applied Research and Technical Development, Fondazione IRCCS Istituto Nazionale dei Tumori, 20133 Milano, Italy; Maurizio.Callari@cruk.cam.ac.uk (M.C.); matteo.dugo@istitutotumori.mi.it (M.D.); patrizia.miodini@istitutotumori.mi.it (P.M.); silvia.veneroni@istitutotumori.mi.it (S.V.); 2 CRUK Cambridge Institute, University of Cambridge, Cambridge CB2 0RE, UK; 3 Department of Medical Oncology, IRCCS Ospedale San Raffaele, 20132 Milano, Italy; bianchini.giampaolo@hsr.it

**Keywords:** Bilateral breast cancer, local relapses, gene expression profiles, tumor microenvironment, stromal genes, PAM50

## Abstract

Metachronous (MBC) and synchronous bilateral breast tumors (SBC) are mostly distinct primaries, whereas paired primaries and their local recurrences (LRC) share a common origin. Intra-pair gene expression variability in MBC, SBC, and LRC derives from time/tumor microenvironment-related and tumor genetic background-related factors and pairs represents an ideal model for trying to dissect tumor-related from microenvironment-related variability. Pairs of tumors derived from women with SBC (*n* = 18), MBC (*n* = 11), and LRC (*n* = 10) undergoing local-regional treatment were profiled for gene expression; similarity between pairs was measured using an intraclass correlation coefficient (ICC) computed for each gene and compared using analysis of variance (ANOVA). When considering biologically unselected genes, the highest correlations were found for primaries and paired LRC, and the lowest for MBC pairs. By instead limiting the analysis to the breast cancer intrinsic genes, correlations between primaries and paired LRC were enhanced, while lower similarities were observed for SBC and MBC. Focusing on stromal-related genes, the ICC values decreased for MBC and were significantly different from SBC. These findings indicate that it is possible to dissect intra-pair gene expression variability into components that are associated with genetic origin or with time and microenvironment by using specific gene subsets.

## 1. Introduction

In the last decades efforts done to guide treatment decision in the adjuvant setting or to predict breast cancer recurrence have been mainly focused on the development of distant metastases [1]. However, the diagnosis of a breast primary tumor is not only associated with life-threatening risk of spread to distant organs, but also with an increased risk of developing contralateral tumors and local recurrence [2,3].

Contralateral breast cancer is regarded as a new primary; it is in fact the most common second primary cancer in women diagnosed with breast cancer and it is not associated to the type of surgery (radical versus breast conserving) [4,5,6,7]. Metachronous bilateral cancers (MBC) occur in 5–10% of patients, while synchronous bilateral cancers (SBC) are rarer, but the incidence rates reported in the literature present a wide range due to the use of different cutoff times to define a contralateral tumor as synchronous. Diagnostic rules allowing for discrimination between multiple primaries and metastatic bilateral lesions are mainly based on histopathological features. The presence of an in situ component, a different histotype and a higher degree of differentiation, are often seen as proofs of a distinct origin. When such criteria do not give a definitive answer, the absence of nodal or distant metastases, and a long time interval tend to support the independent origin of the contralateral tumor [8,9,10]. Despite a set of criteria being established for a diagnostic distinction between contralateral second breast cancer and metastatic spread to the contralateral breast, sometimes it is still difficult to reach a clear diagnostic assessment. Such diagnostic difficulties justify the constant appearance of reports, mainly based on genetic criteria, which exploit new cutting-edge techniques to define the differences or similarities between bilateral primaries [11,12,13,14,15]. These type of studies are important since distinction between new primary and metastasis is not purely a classification issue, but has prognostic and treatment implications. Second primaries would in fact be treated by surgery and radiation (loco-regional treatment) sparing a systemic treatment if reputed not necessary based on clinic-pathological characteristics of the tumor. On the contrary, if considered metastatic, a systemic treatment could be the only type of treatment taken into consideration.

However, in locally recurrent cancer (LRC), the recurrence may be hardly regarded as a second tumor. LRC are more frequently observed in women treated with breast conserving surgery and, although in the major controlled clinical trials no differences in survival rates after conservative versus radical mastectomy have been reported, local recurrence itself is still associated with poorer survival rates [16,17,18]. From a practical point of view local recurrences could be regarded as the consequence of incomplete tumor cells removal during surgery, however differences in the host physiology also play a role, by modifying the original tumor cell biology and impacting on the temporal lapse between diagnosis of the primary tumor and appearance of the recurrence. According to a more recent hypothesis, local recurrences could also be regarded as one of the effects triggered by the phenomenon of self-seeding so far demonstrated only in preclinical models, but giving account of many clinical observations [19,20].

Paired primaries with their local recurrences and tissue samples from women with MBC represent tumors occurring in the same patient, but separated by a time interval; they share therefore as a common leitmotiv, the time factor that is potentially able to modify tumor features due to the interaction with the host, with the tumor microenvironment and with time-related changes. Conversely, under this perspective, synchronous bilateral tumors would represent pairs of tumors where the different genetic background is not further altered by variables linked to time. In other words, paired samples from SBC should differ based only on different clonal derivation, while in MBC, time-induced effects should be added to differences due to their independent origin. On the opposite in LRC, paired samples should share the genetic background and differences should mainly be a consequence of time effects on gene expression. Furthermore, it may be assumed that for tumors with a similar genetic background (i.e., tumor pairs in LRC) the time factor mostly reflects host-induced and tumor microenvironment-related variations.

As a consequence a comparative analysis of gene expression profiles (GEP) between matched primaries/local recurrences and bilateral tumors (both metachronous as well as synchronous) could help to dissect biological differences that are caused by an independent origin from that simply caused by host related time factors. In attempting such “dissection”, we hypothesized that it is possible to identify a list of distinct genes more directly related to time/host factors, and lists of time-independent genes more strictly reflecting the tumor genetic identity, helping this way to distinguish tumor-related from microenvironment-related differences in LRC, MBC, and SBC tumor pairs.

Here, we investigated gene expression variations within three sets of samples, including 18 SBC, 11 MBC, and 10 LRC matched with their corresponding primary tumors. Gene variations between paired samples were compared in the three groups using the intraclass correlation coefficient (ICC), a tool suitable for objectively estimating the degree of similarity. We demonstrate that it is possible to dissect intrapair gene expression variability into components due to genetic origin and time.

## 2. Results and Discussion

### 2.1. Patient and Tumor Characteristics

The study included three series of paired primary tumors/synchronous or metachronous breast lesions from 18 women with SBC and 11 women with MBC and from 10 women with paired primary tumors and local recurrences. Table 1 reports the clinical and pathological characteristics of the three groups of paired samples. Women with SBC were mostly over 50-years old, while patients with MBC and LRC were younger, consistently with young age being a well known risk factor for local relapse. GEP results might be influenced by age, however when tested by analysis of variance (ANOVA), no statistically significant differences (*p* = 0.24) were observed between the ages in the three groups (Figure S1). Technically reliable GEPs were obtained for all analyzed samples.

A preliminary insight into similarities and differences between samples from the same patient was attempted by classifying the patients based on the ER and ERBB2 status, as defined by gene expression data [21]. Data are reported in Figure S2. In two of the local recurrences, we respectively observed loss of *ER* with concomitant acquirement of *ERBB2* expression in one case, and simply loss of *ER* expression in the other case, a pattern that may be compatible with tumor progression. As most of paired samples showed unchanged ER and ERBB2 status (the majority was classified as ER+/ERBB2-), it was impossible to observe any statistically assessable difference.

### 2.2. Correlation between Paired-Samples by Gene Expression Data

**Figure 1:**
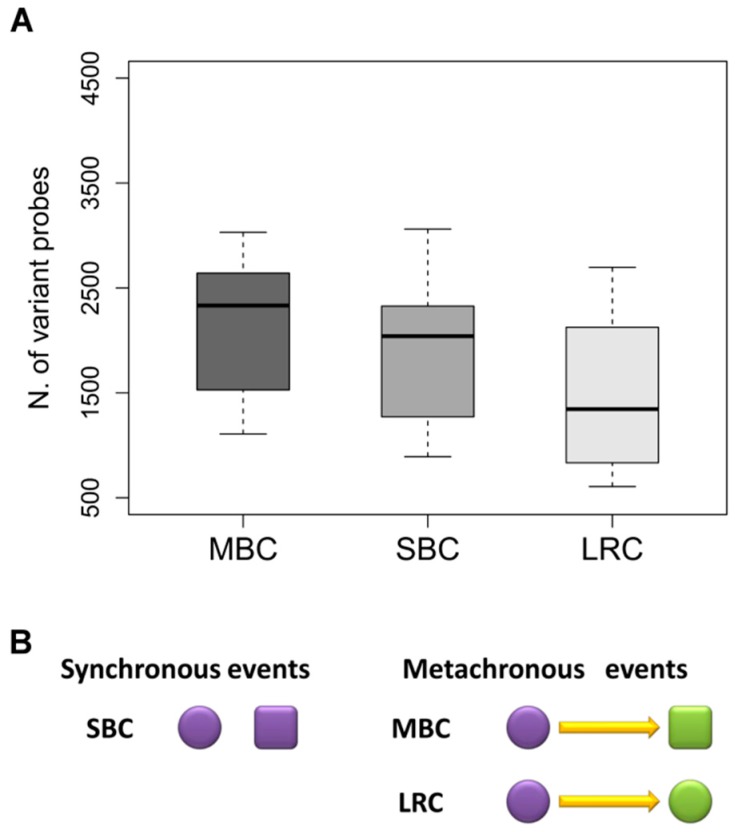
(**A**) Box plots showing the number of probes found to be variantly expressed between tumor pairs from women with metachronous (MBC), synchronous (SBC), and locally relapsed (LCR) breast cancer; (**B**) Schematic representation of sources of variability between pairs from synchronously (SBC) and metachronously (MBC, LRC) diagnosed tumors from the same patient. Shape (square vs. circle) represents genetic background, whereas different color refers to time-related variability. Yellow arrows represent the elapsed time between metachronous events.

Gene expression data allow a more comprehensive comparison between these tumor pairs. For each type of tumor pair, the number of variantly expressed probes having a fold change >2 or <0.5 was measured. The lowest number of variant probes (i.e., 1220 probes) was observed between primaries and their local relapses, whereas the highest number (i.e., 1992 probes) was observed in MBC pairs. Data are shown in Figure 1A.

Indeed, if we consider that the majority of bilateral tumors are distinct primaries and that only a small subset of contralateral tumors represents a metastasis, the variability in gene expression between pairs of MBC could derive from the sum of genetic background-related variability plus host-related effects, whereas in SBC variability would be imputable only to genetic background and in LRC only to time-related/host factors [12,15]. This surely represents a simplification on the sources of variability, which does not take into account the role of many factors, such as anatomic localization, and related vascularization, oxygen/nutrient availability, or even the fact that a local recurrence may contain clones with new mutations. Our hypothesis on the relative contribution of genetic and time-related factors to inter-pair variability in MBC, SBC, and LRC is schematically represented in Figure 1B. Gene expression in tumors is not a static and deterministic entity, simply dictated by the specific combination of gene aberrations that characterize the aberrant genetic background of each tumor, but it is rather a dynamic feature influenced by host-related modifications, such as—among others—hormonal, nutritional, immunological factors.

Gene expression information was used in combination with a correlation-based approach, the ICC, to better assess intra-patient variability in each patient subset [22,23]. With such an approach, which simply measures intra-pair similarity for each probe, it is not necessary to define a primary and secondary lesion within the pairs, making the approach applicable also to SBC. Higher ICC values indicate a higher intra-pair similarity.

**Figure 2:**
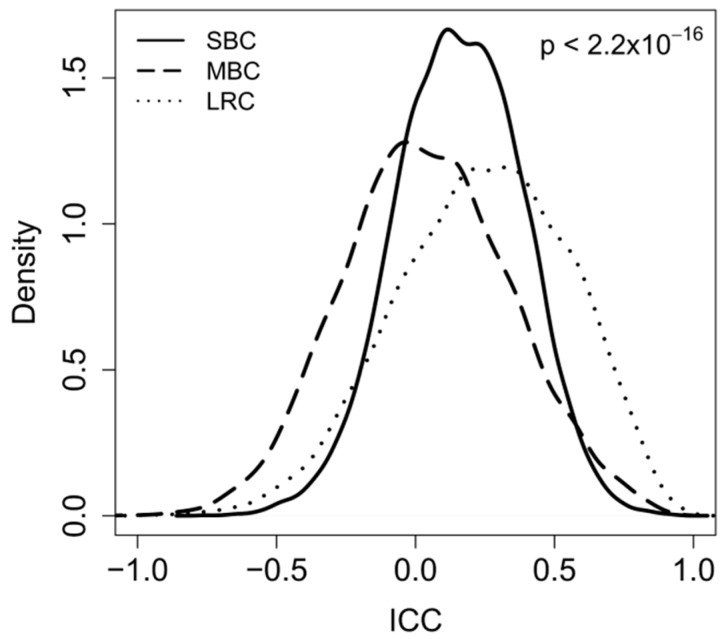
Distribution of intraclass correlation coefficient (ICC) values calculated for each single gene for tumor pairs derived from women with metachronous (MBC), synchronous (SBC), and locally relapsed (LCR) breast cancer. Only the 8586 most variable genes (IQR > 0.5) were used. Differences in the ICC distributions in the three groups (SBC; MBC; and, LRC) were tested using analysis of variance (ANOVA). *p* < 2.2 × 10^−16^.

The initial analysis with the ICC was carried out employing a list of the most variable genes, which were identified by filtering the gene probes by inter quartile range (IQR) and selecting only those with an IQR > 0.5. That way, 8586 highly variable, but biologically unselected probes, were used to estimate how similar the three groups of paired samples (primary/metachronous contralateral tumor; synchronous bilateral tumor pairs, primary/local recurrence) were. As can be seen in Figure 2, the distributions of ICC values for the tested genes were significantly (*p* < 2.2 × 10^−16^) different for the three types of pairs and ranked in a way that mirrored the contribution of one single factor (either time or genetic background) or a combination of the two. Indeed, higher ICC values were observed for pairs in LRC when compared to pairs in both SBC and MBC. By setting for the correlation coefficient a cutoff value of ICC > 0.5, a total number of 1887 genes were above the threshold among the LCR pairs, 597 among the SBC, and 511 among the MBC pairs, suggesting a decrease in intra-pair similarity in contralateral disease when compared to local relapse. The ranking of the median number of differentially expressed DE genes (1221, 1657, 1982, respectively, for LRC, SBC, and MBC), the ICC distributions and the median ICC values (0.25; 0.16; 0.03, respectively, for LRC, SBC, and MBC) were consistently interpreted as a consequence of the different combination of the contributions of time- and genetic background-related variability in the three clinical scenarios.

Based on these premises and on the data shown in Figure 1 and Figure 2, we attempted to find a biologically reasonable interpretation. The pairs with the highest number of correlated genes were primary tumors and their local relapses. In fact, as already stated above, such tumor pairs share a common genetic background and the observed variations in gene expression are probably due to factors related to the elapsed time that was always more than 42 months. Conversely, the genetic background of bilateral tumors is likely to be different as in most cases such tumors are a second primary, and in keeping with this they showed a higher variability between gene expressions. The ICC values were the lowest for the MBC, where the time-related variability adds to the genetic background-related variability. Based on our initial hypothesis, we therefore tried to dissect gene expression variability into time related- and genetic-related variability.

### 2.3. Dissecting Intra-Pair Variability with the Aid Microenvironment-Related and Tumor-Intrinsic Gene Signatures

We hypothesized that using the ICC a biological interpretation on the origin of variability between paired samples derived from SBC, MBC, and LRC can be attempted by choosing for the correlative studies gene lists interrogating specific biological pathways.

Therefore, to address genetic-related variability, we performed the same analyses described above, but focusing on the so-called “intrinsic genes”, which were originally selected as genes with significantly greater variation in expression between different tumors than between paired samples from the same tumor [24]. Since, following its original selection, the list of intrinsic genes was further refined for classification purposes and the so-called PAM50 gene list was generated, here we employed such 50 genes for the analysis [25].

Genes contained in the PAM50 list are mainly related to cell cycle, basal cytokeratins, expression of steroid hormones, and EGFR family members. The way that the intrinsic genes were isolated, together with their biological function lets us suppose them to be little influenced by time- and host-related factors, and instead more by the tumor genetic background.

**Figure 3:**
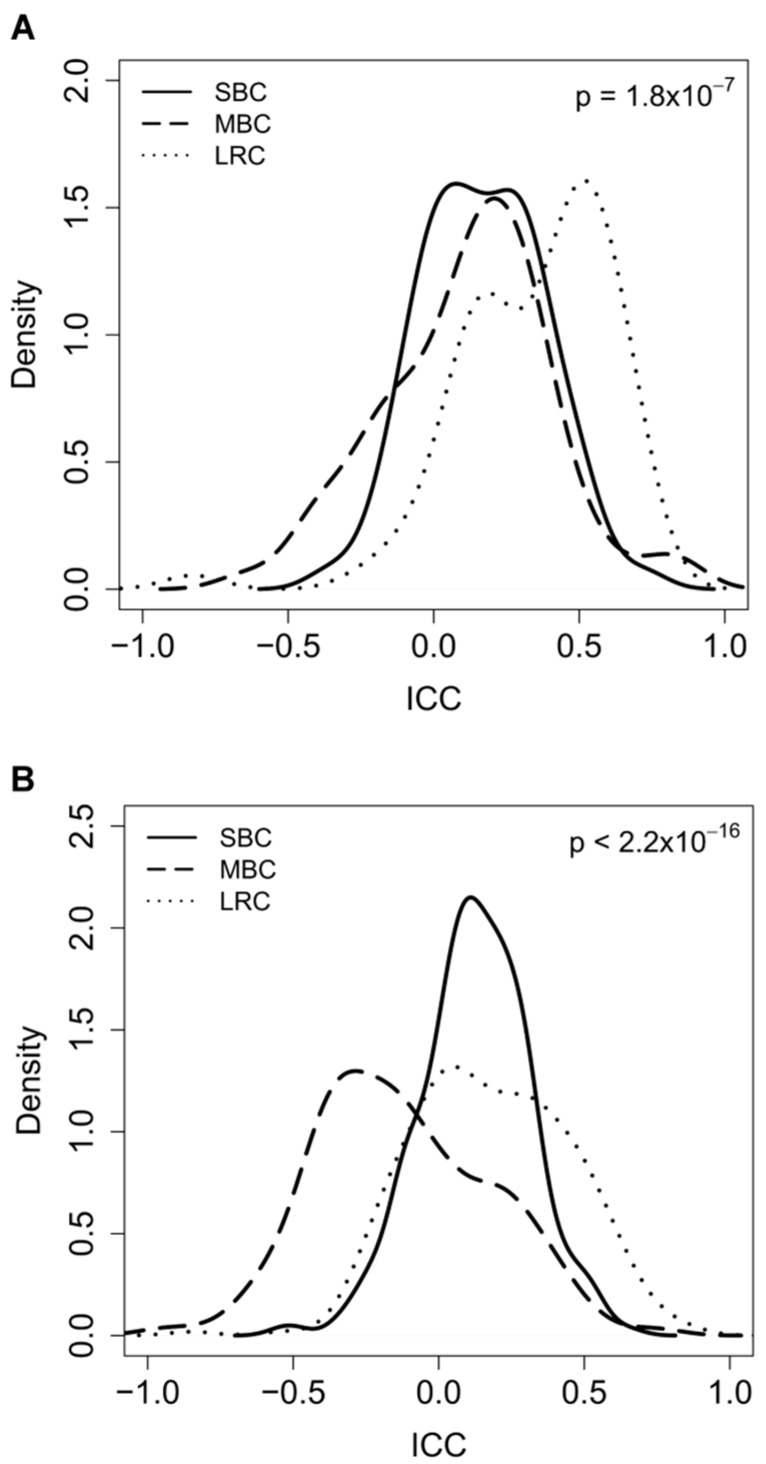
(**A**) Distribution of intraclass correlation coefficient (ICC) values calculated for each single gene form the list of intrinsic genes (PAM50) for tumor pairs derived from women with metachronous (MBC), synchronous (SBC) and locally relapsed (LCR) breast cancer. Differences in the ICC distributions in the three groups (SBC; MBC; and, LRC) were tested using ANOVA; *p =* 1.8 × 10^−7^ (**B**) Distribution of intraclass correlation coefficient (ICC) values calculated for each single gene from a list of stromal genes for tumor pairs derived from women with metachronous (MBC), synchronous (SBC) and locally relapsed (LCR) breast cancer. Differences in the ICC distributions in the three groups (SBC; MBC; and, LRC) were tested using ANOVA. *p* < 2.2 × 10^−16^.

Distributions of ICC values for intrinsic genes are reported in Figure 3A. ICC values for MBC increased when compared to those obtained with the list of unselected genes (median ICC 0.15 vs. 0.03, *p* = 0.03), and an almost complete overlap between MBC and SBC was observed, clearly demonstrating that focusing on such genes eliminates the time-related component of the intra-pair variability. This was further supported by the ICC distribution for LRC, which also shifted towards higher values (median ICC 0.25 vs. 0.41, *p* = 7.0 × 10^−4^). Box-plots comparing the ICC values in MBC, SBC and LRC obtained when using unselected or intrinsic genes are reported in Figure S3A. The observed statistically significant difference (*p* = 1.8 × 10^−7^) between the distributions of ICC values for LRC and contralateral tumors is consistent with the former sharing a common genetic background and the latter being independent primaries.

To further dissect time-and microenvironment-related from genotype-related gene variations, ICC values were also calculated for a set of stroma-related genes [26]. Such genes have been identified comparing gene expression profiles of pairs of fine needle (stroma-poor) and core needle (stroma rich) biopsies of 37 breast tumors. The probesets with higher expression levels in core biopsies when compared to fine needle biopsies were defined as “stromal genes”. The list contains genes coding for immunoglobulins and antigen presentation proteins, as well as for extracellular matrix proteins. As expression of these genes is mainly imputable to the host, as well as to host-tumor interaction and not to the tumor itself, gene expression variations resulting from a different genotype/origin of the tumor should be minimized. In fact, as expected, there was a decrease of ICC values for MBC (both when compared to those obtained with unselected genes as well with intrinsic genes), which shifted to the left and appeared to be significantly different (*p* = 2.2 × 10^−16^) from SBC. This underlines that time related variability is amplified using this gene list. Similarly, and in spite of their common genetic origin, ICC values for LRC also shifted towards lower values (0.25 vs. 0.17 *p* = 4.22 × 10^−4^). Box-plots comparing ICC values in tumor pairs from women with MBC, SBC, and LRC obtained by using unselected versus stromal genes are reported in Figure S3B.

## 3. Material and Methods

### 3.1. Case Series

Fresh frozen primary tumors were collected from patients with operable breast cancer undergoing radical or conservative surgery at the Istituto Nazionale Tumori of Milano (INTM) between 1983 and 1998. Axillary lymph node dissection was performed for all of the cases and only women with pathologically node negative tumors subjected only to local-regional treatment until relapse/new disease manifestation and for whom frozen samples from both breast lesions were available, were included in the study. Bilateral tumors were defined as synchronous when the two tumors were diagnosed within six months time lapse (18 cases), and metachronous if more than 6 months have passed between the two diagnoses (11 cases). Local recurrences obtained at surgical radicalization in women for whom a node negative frozen primary was available were also included in the study (10 cases). For the latter samples margin status was carefully assessed for the invasive and for the in situ component. None of the patients have previously received any type of cancer-related systemic treatment.

Tissue was obtained from leftover frozen samples after routine diagnostic procedures, and was checked for tumor cell content performing an H&E staining on an adjacent tissue section. Only samples containing more than 70% of tumor cells were further processed. A signed consensus form for use of leftover material for research purposes was obtained from all the patients and the study was approved by the IRB and Ethics Committee of INTM.

### 3.2. RNA Extraction and Gene Expression Profiling

Tissue was pulverized using a Mikrodismembrator (Braun Biotech International, Melsungen, Germany) and total RNA was extracted with the Trizol reagent (Invitrogen, Carlsbad, CA, USA) according to manufacturer’s instructions. An additional DNase digestion was performed using the RNeasy kit (Qiagen, Valencia, CA, USA). After each extraction a small fraction of RNA was used for quality and yield assessment. RNA total concentration and purity were determined by UV spectrometry using the NanoDrop2000c (Thermoscientific, Waltham, MA, USA) and the RNA electrophoretic profile was analysed by the Agilent RNA 6000 NanoLabChip kit on the Agilent 2100 Bioanalyzer (Agilent Technologies, Palo Alto, CA, USA) using the software provided by the manufacturer for determination of RIN (RNA integrity number) [27].

RNA samples were processed for microarray hybridization by the Functional Genomics core facility at the Fondazione IRCCS INTM. Briefly, 750 ng of total RNA were reverse transcribed, labeled with biotin and amplified overnight (14 h) using the Illumina RNA TotalPrep Amplification kit (Ambion, Austin, TX, USA), according to manufacturer’s protocol. One µg of the biotinylated cRNA sample was mixed with the Hyb E1 hybridizatioin buffer containing 37.5% (*w/w*) formamide and then hybridized to Illumina HumanWG-6 v3.0 expression beadchip (Illumina, Inc., San Diego, CA, USA) at 58 °C overnight (18 h). The array represents 48,803 bead types, each with a unique sequence derived from human genes in the National Centre for Biotechnology Information Reference Sequence and UniGene database. Array chips were washed with manufacturer’s E1BC solution, stained with 1 µg/mL Cy3-streptavidine (Amersham Biosciences, Little Chanfont, UK) and eventually scanned with Illumina BeadArray Reader (Illumina, San Diego, CA, USA). 

### 3.3. Data Analysis

Array processing—Raw data were obtained from scanned images using the Illumina BeadStudio software (version 3.3.8, Illumina, Inc., San Diego, CA, USA) and pre-processed using the *lumi* package of the Bioconductor project. After quality control, data were normalized using the Robust Spline Normalization method and undetected probes (detection *p*-value > 0.01 in all of the samples) were filtered out. Microarray data were deposited at the Gene Expression data repository (GSE27531).

Gene lists identification—Using the gene symbol as a matching criteria, 73 probes detecting 49 of the 50 intrinsic genes reported by Parker et al. [25] were found in our dataset. The stromal gene signature [26] was similarly identified; of the 206 distinct genes in the signature (detected by 293 Affymetrix probesets), 184 genes were detected in our data by 269 different probes. 

Intraclass Correlation Coefficient—As a measure of similarity of the expression profile of matched samples from the same patient an agreement intraclass correlation coefficient (ICC) was used, which is the ratio of the between-patients variance by the sum of the between-patients, the within-patients and the residual variance as implemented in the *psy* package of the CRAN (cran.r-project.org/) project [22,23]. The ICC value was computed for each probe and distributions of the ICC values for all of the tested genes were evaluated. Differences in the ICC distributions in the three groups (SBC, MBC, and LRC) were tested using ANOVA while a classical *t*-test was applied for two-class comparisons. All of the analyses were performed using R (version 2.10.1).

Receptor status—Estrogen receptor (ER) and ERBB2 status were defined using the expression levels of the respective probes. The threshold value was selected according to the strong bimodal distribution observed [21].

## 4. Conclusions

In patient with SBC, MBC, and LRC the objective estimation of the variability in gene expression within tumor pairs with respect to the global variability allows for drawing interesting biological conclusions. To our knowledge, contralateral tumors have never been considered from this point of view, as most studies were focused on genetic features to understand if the two tumors had a common or distinct genetic origin in order to plan the appropriate type of treatment. We have instead hypothesized that it is possible to dissect intra-pair gene expression variability into components due to genetic origin and to time/host by using appropriate gene lists. Our results confirm this hypothesis and show that tumor instrinsic genes and stromal genes are suitable for such a purpose.

Stroma plays an important role in tumor initiation, progression, and metastatic dissemination [28,29], it is modified at transcriptional level upon interaction with tumor cells and certain stromal-genes may act as early sensors of the transforming process [30]. In addition, age-related stromal changes due to senescence processes occurring in the fibroblasts have also been reported in patients with breast cancer [31]. This indirectly suggests that evaluation of non-microdissected breast tumors looking only at stromal genes, could summarize time-related changes, as was the case in our study.

The bilateral cancer model allowed for us to confirm that intrinsic genes are not influenced by host and time related factors, as expected for genes used for classification purposes. However, since the patient prognosis is not only predicted by molecular subtypes, and since the interaction with the microenvironment is well-known to influence disease outcome [29,32], the clinical application of such genes might be limited by the lack of information on the host. It clearly appears that by including stromal genes, additional gene expression variability (due to host interaction) is captured. 

The possibility to separately analyze host-related and tumor-related gene expression variability is instrumental for a better interpretation of the role of tumor microenvironment and may potentially help to obtain better prediction of prognosis by combining the two types of information.

Studying gene expression profiles may therefore represent a so-far not yet explored approach for better understanding the biology of bilateral tumors, which proved to be valuable despite the low number of studied tumor pairs. On the contrary, molecular genetics approaches, as comparative genomic hybridization (CGH) or next generation sequencing (NGS) are better suitable for distinguishing if bilateral tumors are clonally-related or are distinct primaries [13,15].

## Figures and Tables

**Table 1:** Patient characteristics.

	SBC (*n* = 18 Pairs)		MBC (*n* = 11 Pairs)		LRC (*n* = 10 Pairs)	
Age (years)						
Range	29–82		38–68		30–70	
Median	63.5		57		54.5	
≤50	2 (11.1%)		5 (45.5%)		5 (50%)	
>50	16 (88.9%)		6 (54.5%)		5 (50%)	
Size (cm)	Right	Left	Primary	Secondary	Primary	Secondary
Range	1–8.5	1.2–3.1	1.3–3.7	0.4–3	0.7–2.8	0.5–3.4
Median	2.25	2	1.8	1.7	1.75	1.6
≤2	10 (55.6%)	11 (61.1%)	8 (72.7%)	8 (72.7%)	6 (60%)	6 (60%)
>2	8 (44.4%)	7 (38.9%)	3 (27.3%)	3 (27.3%)	4 (40%)	4 (40%)
ER	Right	Left	Primary	Secondary	Primary	Secondary
positive	12 (66.7%)	15 (83.3%)	10 (90.9%)	10 (90.9%)	10 (100%)	8 (80%)
negative	6 (33.3%)	3 (16.7%)	1 (9.1%)	1 (9.1%)	0	2 (20%)
ERBB2	Right	Left	Primary	Secondary	Primary	Secondary
positive	1 (5.6%)	2 (11.1%)	1 (9.1%)	1 (9.1%)	1 (10%)	2(20%)
negative	17 (94.4%)	16 (88.9%)	10 (90.9%)	10 (90.9%)	9 (90%)	8 (80%)
Time to 2nd event (months)						
Range	-		9–149		42–151	
Median	-		76		74	

SBC: synchronous breast cancer; MBC: metachronous breast cancer; LRC: locally recurrent cancer.

## Supplementary Materials

Supplementary materials can be found at www.mdpi.com/1422-0067/19/1/196/s1.

## References

[1] Harris L.N., Ismaila N., McShane L.M., Andre F., Collyar D.E., Gonzalez-Angulo A.M., Hammond E.H., Kuderer N.M., Liu M.C., Mennel R.G. (2016). Use of Biomarkers to Guide Decisions on Adjuvant Systemic Therapy for Women With Early-Stage Invasive Breast Cancer: American Society of Clinical Oncology Clinical Practice Guideline. J. Clin. Oncol..

[2] Narod S.A. (2014). Bilateral breast cancers. Nat. Rev. Clin. Oncol..

[3] Van der Leij F., Elkhuizen P.H., Bartelink H., van de Vijver M.J. (2012). Predictive factors for local recurrence in breast cancer. Semin. Radiat. Oncol..

[4] Pomerantz R.A., Murad T., Hines J.R. (1989). Bilareal breast cancer. Am. Surg..

[5] Kurtz M., Amalric R., Brandone H., Ayme Y., Spitalier J.M. (1988). Contralateral breast cancer and other second malignancies in patients treated by breast-conserving therapy with radiation. Int. J. Radiat. Oncol. Biol. Phys..

[6] Harvey E.B., Brinton L.A. (1985). Second cancer following cancer of the breast in Connecticut, 1935–82. Nat. Cancer Inst. Monogr..

[7] Rasmussen C.B., Kjær S.K., Ejlertsen B., Andersson M., Jensen M.B., Christensen J., Langballe R., Mellemkjær L. (2014). Incidence of metachronous contralateral breast cancer in Denmark 1978–2009. Int. J. Epidemiol..

[8] Banelli B., Casciano I., Di Vinci A., Gatteschi B., Levaggi A., Carli F., Bighin C., Salvi S., Allemanni G., Ghiorzo P. (2010). Pathological and molecular characteristics distinguishing contralateral metastatic from new primary breast cancer. Ann. Oncol..

[9] Chaudary M.A., Millis R.R., Hoskins E.O., Halder M., Bulbrook R.D., Cuzick J., Hayward J.L. (1984). Bilateral primary breast cancer: A prospective study of disease incidence. Br. J. Surg..

[10] Rubino C., Arriagada R., Delaloge S., Lê M.G. (2010). Relation of risk of contralateral breast cancer to the interval since the first primary tumour. Br. J. Cancer.

[11] Alkner S., Tang M.H., Brueffer C., Dahlgren M., Chen Y., Olsson E., Winter C., Baker S., Ehinger A., Rydén L. (2015). Contralateral breast cancer can represent a metastatic spread of the first primary tumor: Determination of clonal relationship between contralateral breast cancers using next-generation whole genome sequencing. Breast Cancer Res..

[12] Klevebring D., Lindberg J., Rockberg J., Hilliges C., Hall P., Sandberg M., Czene K. (2015). Exome sequencing of contralateral breast cancer identifies metastatic disease. Breast Cancer Res. Treat..

[13] Bao L., Messer K., Schwab R., Harismendy O., Pu M., Crain B., Yost S., Frazer K.A., Rana B., Hasteh F. (2015). Mutational Profiling Can Establish Clonal or Independent Origin in Synchronous Bilateral Breast and Other Tumors. PLoS ONE.

[14] Song F., Li X., Song F., Zhao Y., Li H., Zheng H., Gao Z., Wang J., Zhang W., Chen K. (2015). Comparative genomic analysis reveals bilateral breast cancers are genetically independent. Oncotarget.

[15] Begg C.B., Ostrovnaya I., Geyer F.C., Papanastasiou A.D., Ng C.K.Y., Sakr R.A., Bernstein J.L., Burke K.A., King T.A., Piscuoglio S. (2017). Contralateral breast cancers: Independent cancers or metastases?. Int. J. Cancer.

[16] Fisher B., Anderson S., Bryant J., Margolese R.G., Deutsch M., Fisher E.R., Jeong J.H., Wolmark N. (2002). Twenty-year follow-up of a randomized trial comparing total mastectomy, lumpectomy, and lumpectomy plus irradiation for the treatment of invasive breast cancer. N. Engl. J. Med..

[17] van Dongen J.A., Voogd A.C., Fentiman I.S., Legrand C., Sylvester R.J., Tong D., van der Schueren E., Helle P.A., van Zijl K., Bartelink H. (2000). Long-term results of a randomized trial comparing breast-conserving therapy with mastectomy: European Organization for Research and Treatment of Cancer 10,801 trial. J. Natl. Cancer Inst..

[18] Veronesi U., Cascinelli N., Mariani L., Greco M., Saccozzi R., Luini A., Aguilar M., Marubini E. (2002). Twenty-year follow-up of a randomized study comparing breast-conserving surgery with radical mastectomy for early breast cancer. N. Engl. J. Med..

[19] Kim M.Y., Oskarsson T., Acharyya S., Nguyen D.X., Zhang X.H., Norton L., Massagué J. (2009). Tumor self-seeding by circulating cancer cells. Cell.

[20] Comen E., Norton L. (2012). Self-seeding in cancer. Recent Results Cancer Res..

[21] Callari M., Musella V., Di Buduo E., Sensi M., Miodini P., Dugo M., Orlandi R., Agresti R., Paolini B., Carcangiu M.L. (2014). Subtype-dependent prognostic relevance of an interferon-induced pathway metagene in node-negative breast cancer. Mol. Oncol..

[22] Shrout P.E., Fleiss J.L. (1979). Intraclass correlations: Uses in assessing rater reliability. Psychol. Bull..

[23] Koo T.K., Li M.Y. (2016). A Guideline of Selecting and Reporting Intraclass Correlation Coefficients for Reliability Research. J. Chiropr. Med..

[24] Perou C.M., Sorlie T., Eisen M.B., van de Rijn M., Jeffrey S.S., Rees C.A., Pollack J.R., Ross D.T., Johnsen H., Akslen L.A. (2000). Molecular portraits of human breast tumours. Nature.

[25] Parker J.S., Mullins M., Cheang M.C., Leung S., Voduc D., Vickery T., Davies S., Fauron C., He X., Hu Z. (2009). Supervised risk predictor of breast cancer based on intrinsic subtypes. J. Clin. Oncol..

[26] Bianchini G., Qi Y., Alvarez R.H., Iwamoto T., Coutant C., Ibrahim N.K., Valero V., Cristofanilli M., Green M.C., Radvanyi L. (2010). Molecular anatomy of breast cancer stroma and its prognostic value in estrogen receptor-positive and-negative cancers. J. Clin. Oncol..

[27] Schroeder A., Mueller O., Stocker S., Salowsky R., Leiber M., Gassmann M., Lightfoot S., Menzel W., Granzow M., Ragg T. (2006). The RIN: An RNA integrity number for assigning integrity values to RNA measurements. BMC Mol. Biol..

[28] Kalluri R., Zeisberg M. (2006). Fibroblasts in cancer. Nat. Rev. Cancer.

[29] Joyce J.A., Pollard J.W. (2009). Microenvironmental regulation of metastasis. Nat. Rev. Cancer.

[30] Merlino G., Miodini P., Paolini B., Carcangiu M.L., Gennaro M., Dugo M., Daidone M.G., Cappelletti V. (2016). Stromal Activation by Tumor Cells: An in Vitro Study in Breast Cancer. Microarrays (Basel).

[31] Brouwers B., Fumagalli D., Brohee S., Hatse S., Govaere O., Floris G., Van den Eynde K., Bareche Y., Schöffski P., Smeets A. (2017). The footprint of the ageing stroma in older patients with breast cancer. Breast Cancer Res..

[32] Merlino G., Miodini P., Callari M., D’Aiuto F., Cappelletti V., Daidone M.G. (2017). Prognostic and functional role of subtype-specific tumor–stroma interaction in breast cancer. Mol. Oncol..

