# Supplementary material for: Dissecting Time- from Tumor-Related Gene Expression Variability in Bilateral Breast Cancer
Source: Int J Mol Sci. 2018 Jan 9;19(1):196. doi: 10.3390/ijms19010196 (PMC5796145; doi:10.3390/ijms19010196)
Supplement: Supplementary file 1 [file ijms-19-00196-s001.pdf]

Supplementary Materials: Dissecting Time- from Tumor-Related Gene Expression Variability in Bilateral Breast Cancer

Maurizio Callari, Matteo Dugo, Patrizia Miodini, Silvia Veneroni, Giampaolo Bianchini, Maria Grazia Daidone and Vera Cappelletti

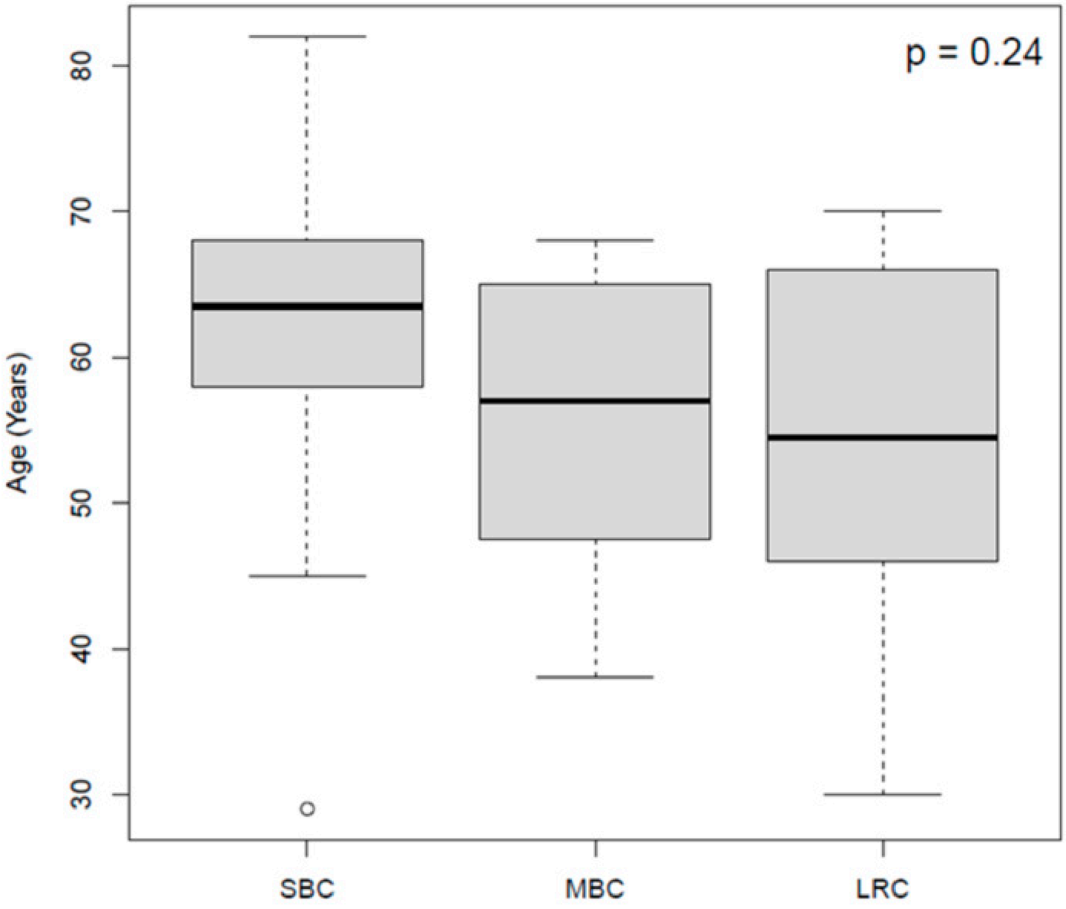

Figure S1. Box plots reporting the ages in the three case series SBC: synchronous breast cancer; MBC: metachronous breast cancer; LRC: locally recurrent cancer. $p = 0.24$.

A

LEFT TUMOR

RIGHT TUMOR	ER+ ERBB2+	ER+ ERBB2-	ER- ERBB2+	ER- ERBB2-
ER+ ERBB2+	-	-	-	-
ER+ ERBB2-	-	10	2	-
ER- ERBB2+	-	1	-	-
ER- ERBB2-	-	4	-	1

B

CONTRALATERAL TUMOR

PRIMARY TUMOR	ER+ ERBB2+	ER+ ERBB2-	ER- ERBB2+	ER- ERBB2-
ER+ ERBB2+	1	-	-	-
ER+ ERBB2-	-	8	-	1
ER- ERBB2+	-	-	-	-
ER- ERBB2-	-	1	-	-

C

LOCAL RECURRENCE

PRIMARY TUMOR	ER+ ERBB2+	ER+ ERBB2-	ER- ERBB2+	ER- ERBB2-
ER+ ERBB2+	-	-	1	-
ER+ ERBB2-	-	8	1	-
ER- ERBB2+	-	-	-	-
ER- ERBB2-	-	-	-	-

Figure S2. Classification of tumor pairs by ER and ERBB2 status as defined by gene expression data for women with synchronous bilateral (panel **A**), metachronous bilateral (MBC) (panel **B**), and locally relapsed (panel **C**) breast cancer.

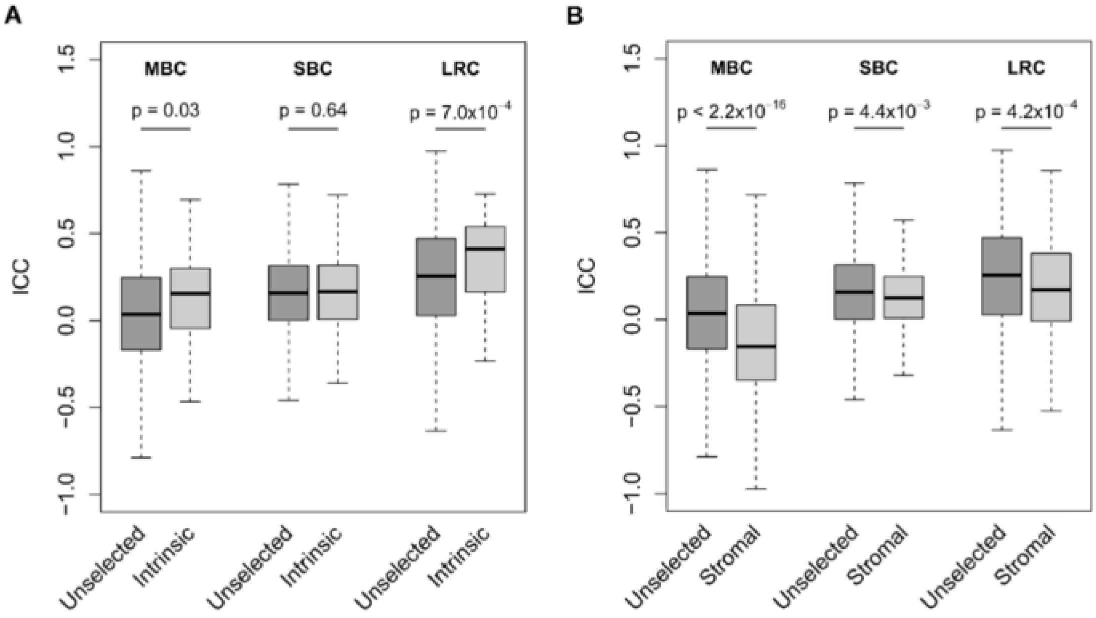

Figure S3. Panel **A.** Box-plots reporting intraclass correlation coefficient (ICC) values obtained for tumor pairs derived from women with metachronous (MBC), synchronous (SBC) and locally relapsed (LCR) breast cancer, when using unselected genes (filtered by IQR) or intrinsic genes. Panel **B**. Box-plots reporting ICC values obtained for tumor pairs derived from women with metachronous (MBC), synchronous (SBC) and locally relapsed (LCR) breast cancer, when using unselected genes (filtered by IQR) or intrinsic genes. *p* values are reported in the Figures.
